# Differences in Lyme Disease Diagnosis among Medicaid and Medicare Beneficiaries, United States, 2016–2021

**DOI:** 10.3201/eid3109.241653

**Published:** 2025-09

**Authors:** L. Hannah Gould, Sarah J. Willis, Christopher G. Prener, Stephanie A. Duench, Holly Yu, Luis Jodar, Jennifer C. Moïsi, James H. Stark

**Affiliations:** Global Vaccines Medical Affairs, Pfizer, Inc., New York, New York, USA (L.H. Gould); Global Vaccines Medical Affairs, Pfizer, Inc., Cambridge, Massachusetts, USA (S.J. Willis, J.H. Stark); RWE Platforms and Partnerships, Pfizer, Inc., New York (C.G. Prener); US Medical Affairs, Vaccines and Anti-infectives, Pfizer, Inc., Collegeville, Pennsylvania, USA (S.A. Duench); Global HTA, Value & Evidence Vaccines, Pfizer, Inc., Collegeville (H. Yu); Global Vaccines Medical Affairs, Pfizer, Inc., Collegeville (L. Jodar); Global Vaccines Medical Affairs, Inc., Pfizer, Paris, France (J.C. Moïsi)

**Keywords:** Lyme disease, vector-borne infections, bacteria, ticks, Borrelia burgdorferi, Medicaid, Medicare, race, ethnicity, United States

## Abstract

Lyme disease is the most common vectorborne disease in the United States. Evidence suggests that persons from racial and ethnic minority groups experience more severe disease. We used a claims-based algorithm on data from 16 jurisdictions with high Lyme disease incidence to identify cases among 4 populations: Medicaid beneficiaries <18 and >19 years of age, and Medicare fee-for-service beneficiaries <65 and >65 years of age. We calculated the prevalence of disseminated disease, hospitalization, and other clinical and epidemiologic parameters by race and ethnicity. We found that non-White persons were more likely than White persons to be female, hospitalized at diagnosis, diagnosed outside of primary care, diagnosed outside of the peak months for Lyme disease transmission, and have disseminated disease. Those data illustrate differences in Lyme disease by race and ethnicity and suggest possible differences across other sociodemographic characteristics. Additional prevention methods are needed to reduce differences in Lyme disease recognition and severity.

Lyme disease is the most reported vectorborne disease in the United States, and ≈476,000 cases are diagnosed and treated annually (*1*,*2*). Lyme disease is caused by infection with the bacterium *Borrelia burgdorferi* sensu lato, which is transmitted to humans by the bite of infected *Ixodes* spp. ticks. Approximately 80% of Lyme disease patients experience an erythema migrans rash at the site of the tick bite (*3*). Without recognition or treatment, the bacteria can disseminate and infect multiple organ systems, resulting in a range of disseminated manifestations that can have neurologic, cardiac, and musculoskeletal presentations (*3*). Although all manifestations are treatable with recommended antimicrobial drugs (*4*), patients with later-stage manifestations are more likely to be hospitalized and to have persistent symptoms after treatment than patients diagnosed with early, localized manifestations (*3*).

In US Lyme disease surveillance data, >90% of cases with reported race are in persons who identify as White (*5*,*6*). That demographic distribution is thought to reflect the populations residing in areas where infected ticks are most common, which tend to be more affluent and educated rural and suburban communities in northeastern and midwestern states that have predominantly White populations (*7*–*9*). However, accumulated evidence has shown that non-White persons, particularly persons who identify as Black or African American, have higher rates of disseminated manifestations of Lyme disease, such as neurologic manifestations and arthritis, than do White persons (*10*–*15*). Higher rates of disseminated manifestations among non-White persons likely are caused in part by difficulty seeing and recognizing erythema migrans rash on darker skin, which can lead to misdiagnosis or delayed or missed diagnoses. Other factors, including differential risk behaviors and knowledge (*16*), likely create and perpetuate the differences in Lyme disease diagnoses (*10*).

Prior studies examining Lyme disease epidemiology by race and ethnicity have been too small to fully disaggregate results by group or have collected data that only enabled comparison of outcomes for White versus non-White persons. Given the heterogeneity of the non-White group, analyses using datasets sufficiently powered to generate incidence estimates and more fully describe disease characteristics and progression by race and ethnic group can improve the characterization of Lyme disease epidemiology.

Medicaid and Medicare are health insurance programs administered by the US Centers for Medicaid and Medicare Services. Medicaid provides healthcare coverage to persons with lower income, and Medicare provides coverage for adults >65 years of age and persons of any age with a qualifying disability, end-stage renal disease, or amyotrophic lateral sclerosis (Lou Gehrig’s disease). Administrative claims data from those programs provide an opportunity to obtain a robust sample to further disaggregate disease outcomes by race and ethnicity. We used administrative claims data to investigate the demographic and clinical characteristics, disease outcomes, and healthcare utilization for Lyme disease cases among Medicaid and Medicare beneficiaries residing in US jurisdictions with high Lyme disease incidence rates.

## Materials and Methods

### Study Design

We conducted a retrospective cross-sectional study using Medicare and Medicaid administrative claims databases covering the period of January 1, 2016–December 31, 2021. The study population included persons residing in high-incidence jurisdictions, which are defined as states with an average Lyme disease incidence of >10 confirmed cases/100,000 population for a period of 3 consecutive years (*5*). The included jurisdictions were the states of Connecticut, Delaware, Massachusetts, Maryland, Maine, Minnesota, New Hampshire, New Jersey, New York, Pennsylvania, Rhode Island, Virginia, Vermont, West Virginia, and Wisconsin and the District of Columbia (*5*). We included cases recorded in Medicaid or Medicare fee-for-service administrative claims databases during the study period that met the inclusion criteria.

### Data Sources

The Medicare and Medicaid databases include enrollment information and adjudicated claims for inpatient care, ambulatory care, and outpatient prescriptions. Data available for facility and professional service claims include the dates and places of service, diagnoses, procedures performed, services rendered, and number of visits for professional services. Data available for outpatient pharmacy claims were the drug dispensed, dispensing date, dose, quantity, and number of therapy days supplied.

### Case Identification and Inclusion Criteria

Inclusion criteria were continuous enrollment in Medicaid or Medicare benefits for >183 days during the study period and residence in a jurisdiction with high Lyme disease incidence at the beginning of each eligible enrollment period; we permitted an enrollment gap of up to 45 days to allow for administrative disruptions in coverage. In addition, for Lyme disease cases, we only included patients who were enrolled for at least 183 days before their Lyme disease diagnosis date. We excluded 15% of cases among Medicaid beneficiaries and 3.4% among Medicare beneficiaries because information on self-reported race/ethnicity was missing. We also excluded beneficiaries with >1 Lyme disease diagnosis during the 183 days before the date they met criteria of the case identification algorithm.

We adapted Lyme disease case identification and classification algorithms from prior studies (*2*,*17*) (Appendix Table 1). In brief, we defined an outpatient Lyme disease case as >1 Lyme disease diagnosis code from the International Classification of Diseases, 10th Revision, Clinical Modification (ICD-10-CM), including A69.20, Lyme disease, unspecified; A69.21, meningitis due to Lyme disease; A69.22, other neurologic disorders in Lyme disease; A69.23, arthritis due to Lyme disease; or A69.29, other conditions associated with Lyme disease. Included case-patients also had >7 days of dispensed oral antibiotics (doxycycline, amoxicillin, azithromycin, or cefuroxime axetil), identified using National Drug Codes, or >1 Healthcare Common Procedure Coding System code for intravenous antibiotics (Appendix Table 2) within 30 days of diagnosis. We defined an inpatient case as a principal Lyme disease diagnosis code (ICD-10-CM, A69.2x) or a principal diagnosis code of a documented objective clinical manifestation of Lyme disease or a tickborne disease transmitted by the same vector (e.g., babesiosis) and a secondary diagnosis code for Lyme disease in the same record per the algorithm. 

We further classified Lyme disease cases as localized or disseminated. For disseminated Lyme disease, we classified cases by neurologic, cardiac, or musculoskeletal manifestations, on the basis of the ICD-10-CM codes in the administrative claims (Appendix Table 3). A person could be counted as having a Lyme disease case >1 time during the study period if >183 days had elapsed since the previous diagnosis and the subsequent case happened in the next calendar year.

### Analyses

We separately conducted analyses for each data source and age group. Thus, we had 4 analytic populations: Medicaid beneficiaries <18 years of age, Medicaid beneficiaries >19 years of age, Medicare beneficiaries <65 years of age (disability group), and Medicare beneficiaries >65 years of age. 

Beneficiaries self-identify their race and ethnicity at enrolment. We combined those variables into a single variable with mutually exclusive categories: White, Black or African American, Hispanic, Asian/Pacific Islander, Native American, and other (i.e., patients who selected other and multiracial for race). We used descriptive statistics to summarize continuous variables by mean and SD and categorical variables by frequency and percentage for the following: demographic data (age, sex, seasonality), clinical characteristics and disease outcomes (disease manifestations, hospitalization, underlying conditions), and healthcare utilization (type of provider who diagnosed Lyme disease; number of sick visits in the 30, 60, and 183 days before Lyme disease diagnosis, antibiotic prescriptions; and laboratory testing within 30 days of Lyme disease diagnosis). We defined sick visits as visits that were not billed with codes for routine health examinations or other preventive reevaluation and management codes. We assessed seasonality by calculating counts and percentage of cases by month and peak Lyme disease season (June–August vs. September–March).

We calculated Lyme disease incidence by race or ethnicity for algorithm-defined Lyme disease overall and for disseminated and localized Lyme disease. We calculated incidence across the entire study period among all patients who had >183 days of continuous enrollment in Medicaid or Medicare as the number of beneficiaries with algorithm-defined Lyme disease per 100,000 person-years.

### Observed Person-Time at Risk for Lyme disease

We defined person-time at risk for Lyme disease from date of eligibility to the first instance of meeting the case ascertainment algorithm or end of continuous follow-up, whichever came first: end of study period, withdrawal from health insurance, or death. For beneficiaries with subsequent Lyme disease cases during the study period, we only used the first case for assessing person-time and calculating incidence rates.

We calculated prevalence ratios (PRs) and 95% CIs by using White persons as the reference group. We considered a PR statistically significant when the 95% CI did not include 1.0. We used *t*-tests to evaluate differences in means for continuous variables and χ^2^ tests to compare the distribution of disseminated disease manifestations between groups and considered p<0.05 statistically significant. We performed all analyses and data management in SAS 9.4 (SAS Institute, Inc., https://www.sas.com) and Databricks version 15.4 (https://docs.databricks.com). 

Because of sample size limitations, we summarized most analyses as White versus non-White. We disaggregated analyses of incidence, risk of developing disseminated disease, and hospitalization at the time of diagnosis by race and ethnic group. Because the study involved data that exist in anonymized structured format and contained no patient personal information, we were not required to have institutional review board approval.

## Results

In Medicaid data from 2016–2021, we identified 33,776 Lyme disease cases among beneficiaries <18 years of age and 30,935 cases among beneficiaries >19 years of age. In Medicare data from 2016–2021, we identified 12,911 Lyme disease cases among beneficiaries <65 years of age and 90,913 among beneficiaries >65 years of age. Most cases were in persons who identified as White, ranging from 85.0% among Medicaid beneficiaries >19 years of age to 96.6% among Medicare beneficiaries >65 years of age (Table 1).

**Table 1:** Number of cases and incidence in a study of differences in Lyme disease diagnosis among Medicaid and Medicare beneficiaries, United States, 2016–2021*

Group	Total no. (%) cases†	Localized Lyme disease				Disseminated Lyme disease		
		No. cases	Person-years	Incidence‡		No. cases	Person-years	Incidence‡
Medicaid, age <18 y	33,776	27,165	37,110,817	73.3		5,798	27,058,337	15.7
White	29,405 (87.1)	24,093	17,461,207	138.1		4,576	17,412,994	26.3
Black, African American	1,244 (3.7)	744	9,611,964	7.8		483	9,611,379	5.0
Asian, Pacific Islander	741 (2.2)	551	2,783,663	19.8		181	2,782,940	6.5
Hispanic	2,063 (6.1)	1,526	6,833,641	22.4		495	6,831,111	7.3
Native American	208 (0.6)	159	268,217	59.3		44	267,950	16.4
Other	115 (0.3)	92	152,126	60.5		19	151,963	12.5
Medicaid, age >19 y	30,935	23,663	25,536,958	92.7		5,380	25,496,013	21.1
White	26,298 (85.0)	20,456	13,156,621	155.6		4,214	13,120,278	32.1
Black, African American	1,461 (4.7)	978	6,188,543	15.8		408	6,187,289	6.6
Asian, Pacific Islander	1,082 (3.5)	813	2,745,246	29.6		212	2,743,918	7.7
Hispanic	1,867 (6.0)	1,253	3,235,335	38.8		500	3,233,589	15.5
Native American	191 (0.6)	133	168,295	79.1		40	168,080	23.8
Other	36 (0.1)	–	–	–		–	–	–
Medicare, age <65 y	12,911	8,204	6,933,720	118.4		3,293	6,923,326	47.6
White	11,845 (91.7)	7,581	5,149,098	147.3		2,930	5,139,283	57.1
Black, African American	513 (4.0)	284	1,267,371	22.4		205	1,267,135	16.2
Asian, Pacific Islander	72 (0.6)	50	105,505	47.4		16	105,440	15.2
Hispanic	293 (2.3)	181	284,434	63.7		84	284,264	29.6
Native American	53 (0.4)	32	32,348	99.0		14	32,306	43.4
Other	135 (1.0)	76	94,964	80.1		44	94,897	46.4
Medicare, age >65 y	90,913	65,298	31,199,802	209.4		16,938	31,087,605	54.5
White	87,831 (96.6)	63,252	27,353,152	231.4		16,201	27,243,943	59.5
Black, African American	1,172 (1.3)	753	2,299,786	32.8		324	2,298,791	14.1
Asian, Pacific Islander	591 (0.7)	410	649,660	63.2		141	649,082	21.7
Hispanic	283 (0.3)	183	357,614	51.2		76	357,380	21.3
Native American	64 (0.1)	45	30,197	149.1		14	30,127	46.5
Other	872 (1.1)	655	509,397	128.7		182	508,283	35.8

*–, sample size too small to calculate rate. †Total cases include all cases of Lyme disease identified during the study period. For incidence rate calculations, we included only the first case during the study period for beneficiaries with subsequent Lyme disease diagnoses. ‡Incidence rate was calculated across the entire study period among all persons who had <183 days of continuous enrollment in Medicaid or Medicare and resided in a high incidence state as follows: incidence = [(number beneficiaries with Lyme disease algorithm-defined Lyme disease)/(observed person-time at risk for Lyme disease)] × 100,000 person-years.

### Incidence

Medicare recipients >65 years of age had the highest overall incidence and highest incidence of localized (209.4/100,000 person-years) and disseminated (54.5/100,000 person-years) disease; Medicare recipients <65 years of age also had high incidence of localized (118.4/100,000 person-years) and disseminated (47.6/100,000 person-years) disease. Medicaid beneficiaries <18 years of age had the lowest incident rates for localized (73.3/100,000 person-years) and disseminated (15.7/100,000 person-years) disease. Across all age and beneficiary groups, incidence of disease was highest among White persons; next highest rates were among persons who identified as Native American, and lowest rates were among persons who identified as Black or African American (Table 1).

### Characteristics of Lyme Disease Cases

We noted marked differences in the characteristics of Lyme disease cases for White and non-White Medicaid and Medicare beneficiaries (Tables 2, 3; Appendix Tables 4–7). In all groups, disseminated disease was more prevalent among non-White persons, particularly among Medicaid beneficiaries <18 years of age (PR 1.77 [95% CI 1.68–1.87]) and >19 years of age (PR 1.57 [95% CI 1.49–1.66]). We saw the highest prevalence of disseminated disease among Black/African American Medicare beneficiaries <65 years of age (42.7%, 294/513) and Black/African American Medicaid beneficiaries <18 years of age (39.1%, 487/1,244) (Appendix Tables 4, 6).

**Table 2:** Clinical characteristics of cases among Medicaid beneficiaries in a study of differences in Lyme disease diagnosis among Medicaid and Medicare beneficiaries, United States, 2016–2021*

Characteristic	Medicaid, age <18 y				Medicaid, age >19 y		
	White, n = 29,405	Non-White, n = 4,371	PR (95% CI) or p value		White, n = 26,298	Non-White, n = 4,637	PR (95% CI) or p value
Disseminated disease†	4,685 (15.9)	1,234 (28.2)	1.77 (1.68–1.87)		4,542 (17.3)	1,259 (27.2)	1.57 (1.49–1.66)
Neurologic	1,248 (26.6)	265 (21.5)	0.00006		2,054 (45.2)	518 (41.1)	0.002
Musculoskeletal	3,353 (71.6)	941 (76.3)	NA		2,299 (50.6)	704 (55.9)	NA
Cardiac	84 (1.8)	28 (2.3)	NA		189 (4.2)	37 (2.9)	NA
Hospitalization at diagnosis	645 (2.2)	188 (4.3)	1.96 (1.67–2.30)		1,124 (4.3)	229 (4.9)	1.56 (1.01–1.33)
Diagnosed outside of primary care‡	4,170 (17.7)	644 (19.1)	1.08 (0.99–1.18)		4,893 (23.2)	824 (23.1)	0.99 (0.92–1.08)
Diagnosed during September–March	6,271 (21.3)	1,204 (27.5)	1.29 (1.23–1.36)		7,351 (28.0)	1,596 (34.4)	1.23 (1.18–1.29)
Sex							
M	16,227 (55.2)	2,301 (52.6)	Referent		12,067 (45.9)	1627 (35.1)	Referent
F	13,178 (44.8)	2,070 (47.4)	1.06 (1.02–1.09)		14,231 (54.1)	3,010 (64.9)	1.20 (1.17–1.23)
Mean Quan-Charlson Comorbidity Index score (SD)	1.1 (0.3)	1.1 (0.2)	0.339		1.7 (1.3)	1.8 (1.5)	0.003
Mean no. visits before diagnosis (SD)							
<30 d before	1.8 (2.5)	2.0 (2.5)	<0.0001		2.7 (3.4)	3.2 (3.6)	<0.0001
<60 d before	3.2 (4.5)	3.4 (4.4)	0.006		4.9 (6.3)	5.6 (6.6)	<0.0001
<183 d before	8.8 (12.8)	8.8 (12.3)	0.8843		13.6 (17.6)	14.3 (17.2)	0.0122
Treatment and testing							
Amoxicillin	16,989 (57.8)	2,576 (58.9)	1.02 (0.97–1.08)		4,128 (15.7)	894 (19.3)	1.22 (1.13–1.32)
Doxycycline	12,708 (43.2)	1,882 (43.1)	1.00 (0.94–1.06)		22,518 (85.6)	3,841 (82.8)	0.96 (0.92–1.00)
Antibody testing§	4,141 (14.1)	923 (21.1)	1.08 (1.07–1.11)		4,019 (15.3)	1,025 (22.1)	1.08 (1.07–1.11)

*Values are no. (%) except as indicated. Reference group = White. NA, not applicable; PR, prevalence ratio. †We used χ^2^ test to compare distribution of disseminated manifestations between White and non-White beneficiaries. ‡Calculated only among claims with known provider type. §Current Procedural Terminology code 86618, *Borrelia burdorferi* antibody.

**Table 3:** Clinical characteristics of cases among Medicare beneficiaries in a study of differences in Lyme disease diagnosis among Medicaid and Medicare beneficiaries, United States, 2016–2021*

Characteristic	Medicare, age <65 y				Medicare, age >65 y		
	White, n = 11,845	Non-White, n = 1,066	PR (95% CI) or p value		White, n = 87,831	Non-White, n = 3,037	PR (95% CI) or p value
Disseminated disease†	3,345 (28.2)	400 (37.5)	1.33 (1.22–1.44)		18,277 (20.8)	817 (26.9)	1.29 (1.22–1.37)
Neurologic	2,155 (64.4)	242 (60.5)	NA		9,808 (53.7)	428 (52.4)	0.055
Musculoskeletal	1,101 (32.9)	–	NA		7,127 (39.0)	344 (42.1)	NA
Cardiac	89 (2.7)	–	NA		1,342 (7.3)	45 (5.5)	NA
Hospitalization at diagnosis	967 (8.2)	109 (10.2)	1.25 (1.04–1.51)		6,325 (7.2)	250 (8.2)	1.14 (1.01–1.29)
Diagnosed outside of primary care‡	4,105 (36.4)	447 (44.3)	1.22 (1.08–1.37)		27,719 (32.6)	1,275 (43.6)	1.34 (1.25–1.43)
Diagnosed during September–March	3,793 (32.0)	377 (35.4)	1.10 (1.01–1.20)		22,316 (25.4)	890 (29.3)	1.15 (1.09–1.22)
Sex							
M	5,199 (43.9)	400 (37.5)	Referent		41,438 (47.2)	1,322 (43.5)	Referent
F	6,646 (56.1)	666 (62.5)	1.11 (1.06–1.17)		46,393 (52.8)	1,715 (56.5)	1.07 (1.04–1.10)
Mean Quan-Charlson Comorbidity Index score (SD)	2.4 (2.0)	2.9 (2.3)	<0.0001		2.6 (2)	2.8 (2.2)	<0.0001
Mean no. visits before diagnosis (SD)							
<30 d before	0.99 (1.5)	1.1 (1.6)	0.111		0.7 (1.2)	0.6 (1.1)	<0.0001
<60 d before	1.8 (2.5)	1.9 (2.7)	0.057		1.2 (1.9)	1.0 (1.7)	<0.0001
<183 d before	4.8 (6.2)	5.2 (6.6)	0.448		3.1 (4.4)	2.6 (4.1)	<0.0001
Treatment and testing							
Amoxicillin	2,297 (19.4)	295 (27.7)	1.43 (1.25–1.64)		13,594 (15.5)	632 (20.8)	1.32 (1.21–1.44)
Doxycycline	9,208 (77.7)	758 (71.1)	0.92 (0.83–1.01)		73,558 (83.7)	2,366 (77.9)	0.92 (0.87–0.97)
Antibody testing§	5,619 (47.4)	522 (49.0)	1.03 (0.97–1.09)		42,212 (48.1)	1,617 (53.2)	1.10 (1.06–1.13)

*Values are no. (%) except as indicated. Reference group = White. NA, not applicable; PR, prevalence ratio; –, sample size <11, thus counts suppressed from calculations. †We used χ^2^ test to compare distribution of disseminated manifestations between White and non-White beneficiaries. ‡Calculated only among claims with known provider type. §Current Procedural Terminology code 86618, *Borrelia burdorferi* antibody.

Musculoskeletal disease (arthritis) was the most common disseminated manifestation among Medicaid beneficiaries of all ages and was more prevalent among non-White than White beneficiaries <18 years of age (941/4371 [76.3%] vs. 3,353/29,405 [71.6%]; p = 0.00006) and >19 years of age (704/4637 [55.9%] vs. 2,299/26,298 [50.6%]; p = 0.002) (Table 2). Among Medicare beneficiaries, neurologic manifestations were the most common disseminated manifestation, but we noted no difference in the prevalence of disseminated manifestations by race or ethnic group among Medicare beneficiaries >65 years of age (Table 3).

Across all age and beneficiary groups, non-White persons had a higher prevalence of hospitalization at diagnosis, particularly among Medicaid beneficiaries <18 years of age (PR 1.96 [95% CI 1.67–2.30]). Non-White persons with Lyme disease were more likely to be female and were more likely to be diagnosed during September–March, outside of the peak months for Lyme disease transmission.

Among Medicaid beneficiaries, we noted no difference between White and non-White persons in the prevalence of diagnosis outside of primary care. Among Medicaid beneficiaries >19 years of age, non-White persons had a higher mean Quan-Carlson Comorbidity Index score than did White persons (Table 2). Among Medicare beneficiaries, non-White persons were more likely to have a diagnosis outside of primary care and had higher mean Quan-Charlson Comorbidity Index scores than were White persons (Table 3).

Non-White beneficiaries also had more sick visits in the 30 and 60 days before Lyme disease diagnosis than did White persons, except among Medicare beneficiaries <65 years of age, for whom we noted no difference. Non-White Medicaid beneficiaries >19 years of age also had more sick visits in the 183 days before Lyme disease diagnosis than did White beneficiaries. Among Medicare beneficiaries >65 years of age, White beneficiaries had more sick visits in the 183 days before Lyme disease diagnosis than did non-White beneficiaries.

Among Medicaid beneficiaries <18 years of age, we noted no differences in the prevalence of receiving an amoxicillin or doxycycline prescription by White versus non-White race/ethnicity. In the other 3 groups, non-White beneficiaries were more likely than White beneficiaries to receive an amoxicillin prescription and less likely to receive a doxycycline prescription.

Among Medicare beneficiaries <65 years of age, we saw no difference in the percentage of persons who received a *B. burgdorferi* antibody test by White versus non-White race/ethnicity. In the other 3 groups, non-White beneficiaries were more likely than White beneficiaries to have had antibody testing.

### Disseminated Disease and Hospitalization by Disaggregated Race and Ethnic Group

Black/African American, Asian/Pacific Islander, and Hispanic Medicaid beneficiaries of all ages and Medicare beneficiaries <65 years of age were more likely than White beneficiaries to have disseminated Lyme disease manifestations (Table 4). Black/African American, Asian/Pacific Islander, and Hispanic pediatric Medicaid beneficiaries were also more likely to be hospitalized than were White beneficiaries. Compared with White Medicaid beneficiaries <18 years of age, Black/African American children had a PR of 2.35 (95% CI 2.15–2.56) for developing disseminated disease and PR of 2.57 (95% CI 1.96–3.36) for hospitalization.

**Table 4:** Prevalence ratios for development of disseminated Lyme disease and hospitalization by race and ethnicity among Medicaid and Medicare beneficiaries, United States, 2016–2021*

Group	Prevalence ratio (95% CI)	
	Disseminated disease	Hospitalization at diagnosis
Medicaid, age <18 y		
Black, African American	2.35 (2.15–2.56)	2.57 (1.96–3.36)
Asian, Pacific Islander	1.57 (1.34–1.83)	1.71 (1.09–2.69)
Hispanic	1.51 (1.37–1.66)	1.59 (1.21–2.11)
Native American	1.16 (0.84–1.60)	1.14 (0.56–2.21)
Other	1.45 (0.91–2.29)	0.74 (0.11–5.20)
Medicaid, age >19 y		
Black, African American	1.67 (1.52–1.84)	1.58 (1.27–1.98)
Asian, Pacific Islander	1.23 (1.06–1.42)	0.87 (0.60–1.26)
Hispanic	1.73 (1.59–1.89)	0.90 (0.69–1.19)
Native American	1.19 (0.85–1.65)	0.51 (0.17–1.56)
Other	1.75 (0.77–3.95)	–
Medicare, age <65 y		
Black, African American	1.51 (1.35–1.68)	1.51 (1.17–1.94)
Asian, Pacific Islander	0.83 (0.53–1.29)	1.53 (0.80–2.93)
Hispanic	1.09 (0.90–1.31)	0.83 (0.53–1.31)
Native American	1.05 (0.68–1.64)	1.30 (0.57–2.99)
Other	1.37 (1.10–1.72)	1.11 (0.63–1.96)
Medicare, age >65 y		
Black, African American	1.46 (1.33–1.61)	1.17 (0.95–1.43)
Asian, Pacific Islander	1.18 (1.01–1.38)	1.06 (0.78–1.44)
Hispanic	1.48 (1.22–1.78)	1.21 (0.80–1.82)
Native American	1.11 (0.67–1.83)	1.87 (0.94–3.73)
Other	1.07 (0.94–1.22)	1.14 (0.90–1.45)

*Reference group White for all comparisons. ­–, sample size too small to calculate.

### Seasonality

Among all groups, June and July were the peak months for Lyme disease diagnosis (Appendix Figure). White persons were more likely than non-White persons to receive a Lyme disease diagnosis during the summer months. Across all groups, Black/African American persons were more likely to receive diagnoses during December–February than during peak Lyme disease months.

## Discussion

Using 2 large administrative datasets that include healthcare claims for nearly 40% of children and half of older adults in the United States (*18*,*19*), this study found substantial differences in Lyme disease diagnoses across race and ethnic groups in the United States. Those differences were most pronounced for children, particularly Black/African American children, who had more than twice the prevalence of more severe, disseminated disease than did White children. Those results expand on prior published literature by identifying group-specific differences in the clinical manifestations and demographic characteristics of persons with Lyme disease in US states with high Lyme disease incidence.

This study provides a demographic profile of Lyme disease cases in high-incidence states that is difficult to discern from US Lyme disease surveillance data, in which race and ethnicity are unknown for nearly 40% of reported cases (*5*). As observed in Lyme disease surveillance data, we saw that cases among White persons are overrepresented compared with their representation in the population. However, that observation was attenuated among Medicaid beneficiaries, likely reflecting the overall demographic distribution of persons covered by that insurance program, which is more racially and ethnically diverse than the US population, particularly among pediatric beneficiaries (*20*). Because US Lyme disease surveillance in high-incidence states is now laboratory-based (*21*), information on race and ethnicity is likely to continue to be underreported because those variables are not routinely available in laboratory reporting systems, highlighting the importance of administrative claims to supplement surveillance data.

Although detailed analyses were limited by the small sample size, we found that incidence rates among Medicaid and Medicare beneficiaries who identified as Native American were second to those of White persons; however, Native American persons did not have increased risk for disseminated disease or hospitalization. Medicaid provides health coverage to ≈30% of Native American/Alaska Native persons <65 years of age in the eastern United States and nationally to ≈60% of Native American/Alaska Native children (*22*,*23*). Future analyses on the epidemiology of Lyme disease in Native American populations could also include Indian Health Service data. Regardless, recognizing the seemingly high incidence of Lyme disease in that population, jurisdictions in high Lyme disease–incidence states with substantial Native American populations might consider routine analysis of Lyme disease outcomes by race and ethnic group and develop tailored Lyme disease education programs and interventions (*24*).

Diagnosis of disseminated Lyme disease might indicate that early signs and symptoms were missed or not recognized as Lyme disease (*3*). Diagnostic differences were most pronounced among persons who identified as Black or African American, suggesting that difficulties in recognition of the erythema migrans rash on darker skin likely account for at least some of those differences, as previously suggested (*10*). However, the finding that non-White beneficiaries also had more sick visits in the 30 and 60 days before their Lyme disease diagnosis than did White beneficiaries suggests that patients, especially Medicaid beneficiaries, might have sought care frequently enough to provide opportunity to diagnose Lyme disease. Such patient journeys have been described in case reports (*25*,*26*). In addition, reports have documented that persons who identify as Black/African American or Hispanic receive lower quality healthcare for multiple diseases and conditions than do persons who identify as White, which can delay diagnosis and lead to inferior outcomes (*27*,*28*).

Musculoskeletal disease (arthritis) was the most common disseminated manifestation among Medicaid beneficiaries and was also a more prevalent disseminated manifestation among non-White Medicaid beneficiaries. Because arthritis represents the late stage of disease dissemination (*3*), that finding further suggests differences in time to disease diagnosis among persons from racial and ethnic minority groups and is consistent with our finding that fewer cases were diagnosed during the summer months among non-White persons, which also has been reported by others (*11*,*16*,*29*,*30*). Differences in the development of disseminated manifestations and seasonality of infections have also been found by age and sex (*31*,*32*). Other possible explanations for those differences include differential care-seeking behaviors and healthcare access for some groups and misdiagnosis, including possible overdiagnosis and treatment of Lyme disease in some groups.

Our findings cannot be readily explained by differences in underlying health status for certain race or ethnic groups. For example, the greatest difference in development of disseminated disease was for Black/African American children, and that population had a similar number of underlying conditions as White children. Conversely, differences in the development of disseminated disease were less pronounced for Native American beneficiaries compared with other racial and ethnic minority groups, and Native American beneficiaries had more underlying conditions than did White beneficiaries. Although limited, some evidence suggests that certain conditions, such as hypercholesterolemia (*33*), might predispose persons to Lyme disease. The higher Charlson Comorbidity Index among some groups, along with more frequent healthcare utilization before diagnosis, also might reflect misdiagnosis of Lyme disease in the face of chronic, unexplained illness.

Of note, we found a high incidence of Lyme disease among beneficiaries of the disability portion of Medicare, which represents ≈12% of total Medicare enrollment. Across all racial groups, about one third of persons with Lyme disease in the disability group had disseminated disease develop, particularly neurologic manifestations, and nearly 10% were hospitalized. That finding has implications for considering risk related to Lyme disease and tick exposure, which extends beyond rigorous outdoor activities to include risk factors around the home and yard (*34*,*35*). In addition, although the high hospitalization rates in that population could be because of a higher prevalence of underlying illness, the high percentage of disseminated manifestations suggests possible delays in diagnosing Lyme disease, because of either a missed rash or conflation with other conditions.

Although claims data are extremely valuable for the efficient and effective examination of healthcare outcomes, treatment patterns, and healthcare resource utilization, those data are collected for the purpose of payment and not research. A validation study of the claims-based algorithms for Lyme disease case identification used in this study found the positive predictive value was 93.8% (95% CI 88.1%–97.3%) for confirmed, probable, or suspected cases and 66.4% (95% CI 57.5%–74.5%) for confirmed and probable cases (*17*). More recent efforts to evaluate the claims-based algorithm suggest that varying algorithm parameters related to the type and timing of antimicrobial therapy might further improve the identification of Lyme disease cases; however, characteristics of Lyme disease diagnoses did not differ greatly between 3 modified case definitions (*36*). On the other hand, if differences in prescribing or coding patterns related to Lyme disease by patient race and ethnicity exist, cases possibly were misclassified or missed using those algorithms. Regardless, misclassification of some Lyme disease cases remains a possibility. Similarly, our classification of disseminated versus localized disease could be incorrect for some cases. In addition, although our classification of sick visits was designed to exclude visits conducted for primary or ongoing routine care, we could not confirm the reason for individual healthcare visits.

Race and ethnicity in the Medicaid and Medicare datasets are self-reported, the standard for ascertainment of those demographic variables, and overall missingness was limited, especially in the Medicare claims database, supporting the overall robustness of the data. However, one limitation of our study is that we cannot rule out misclassification of race and ethnicity or missingness of race and ethnicity for some groups, particularly for persons of Hispanic ethnicity (*37*,*38*); thus, some groups might remain underrepresented or misclassified in our results. Second, although our goal was to disaggregate findings by race/ethnic group, sample sizes were too small to do so for some analyses. Finally, our findings cannot be generalized to populations with other types of insurance coverage.

In conclusion, the incidence of Lyme disease continues to increase in the United States. Our characterization of Lyme disease diagnoses and outcomes by race and ethnicity provides insights into populations most at risk for potential long-term outcomes and highlights imbalances in disease diagnoses. To improve Lyme disease detection and reduce severe disease, healthcare providers who see patients receiving publicly funded insurance need Lyme disease prevention and education programs.

Appendix Additional information differences in Lyme disease diagnosis among Medicaid and Medicare beneficiaries, United States, 2016–2021.
