# Supplementary material for: Differences in Lyme Disease Diagnosis among Medicaid and Medicare Beneficiaries, United States, 2016–2021
Source: Emerg Infect Dis. 2025 Sep;31(9):1755–63. doi: 10.3201/eid3109.241653 (PMC12407116; doi:10.3201/eid3109.241653)
Supplement: Appendix — Additional information differences in Lyme disease diagnosis among Medicaid and Medicare beneficiaries, United States, 2016–2021. [file 24-1653-Techapp-s1.pdf]

Article DOI: https://doi.org/10.3201/eid3109.241653

EID cannot ensure accessibility for supplementary materials supplied by authors. Readers who have difficulty accessing supplementary content should contact the authors for assistance.

Differences in Lyme Disease Diagnosis among Medicaid and Medicare Beneficiaries, United States, 2016–2021

Appendix

Appendix Table 1. Lyme disease case ascertainment and classification algorithms*

Variable	Definition
LD case ascertainment (outpatient)	For outpatient records: At least one LD ICD-CM diagnosis code and at least 7 d of dispensed antibiotics. Dispensed antibiotics and diagnosis codes must be within 30 d of one another. The date of diagnosis will be the earliest of the diagnosis and dispensing dates.
LD case ascertainment (inpatient)	For inpatient records: (1) a principal diagnosis code (A69.2x) or (2) a principal diagnosis code of a documented objective clinical manifestation of LD or a tickborne disease transmitted by the same vector (e.g., babesiosis) and a secondary diagnosis code for LD in the same record per the algorithm from {Schwartz, 2021 #184} and at least seven days of dispensed oral antibiotics (doxycycline, amoxicillin, azithromycin, or cefuroxime axetil), identified using National Drug Codes, or at least one Healthcare Common Procedure Coding System code for intravenous antibiotics within 30 d of diagnosis. Patients meeting both the outpatient and inpatient algorithm on the same day will be classified as inpatient.
Localized LD	Patient records that include only A69.20 will be classified as localized cases.
Disseminated LD	Patient records that meet any one of the following criteria will be classified as disseminated:<br>1) One or more non-A69.20 codes for LD at the time of diagnosis<br>2) A69.20 as well as another non-A69.20 ICD-10 code for LD within 30 d<br>3) A69.20 and one or more of the ICD-10 codes in Appendix Table 3 within 30 d of one another
Neurologic LD	Patients with codes A69.21 or A69.22<br>or<br>Patients with A69.20 code and one or more of the neurologic LD ICD-10 codes in Appendix Table 3 within 30 d of one another
LD Arthritis	Patients with code A69.23<br>or<br>Patients with A69.20 code and one or more of the musculoskeletal LD ICD-10 codes in Appendix Table 3 within 30 d of one another
Cardiac LD	Patients with A69.20 code and one or more of the cardiovascular LD ICD-10 codes in Appendix Table 3 within 30 d of one another

*LD, Lyme disease

Appendix Table 2. Healthcare Common Procedure Coding System codes for intravenous antibiotics included in case ascertainment

Code type	ICD Code	Definition
HCPC	J0530	Injection, penicillin g benzathine and penicillin g procaine, up to 600,000 units
HCPC	J0540	Injection, penicillin g benzathine and penicillin g procaine, up to 1,200,000 units
HCPC	J0550	Injection, penicillin g benzathine and penicillin g procaine, up to 2,400,000 units
HCPC	J0558	Injection, penicillin g benzathine and penicillin g procaine, 100,000 units
HCPC	J0559	Injection, penicillin g benzathine and penicillin g procaine, 2500 units
HCPC	J0560	Injection, penicillin g benzathine, up to 600,000 units
HCPC	J0561	Injection, penicillin g benzathine, 100,000 units
HCPC	J0580	Injection, penicillin g benzathine, up to 2,400,000 units
HCPC	J0696	Injection, ceftriaxone sodium, per 250 mg
HCPC	J0697	Injection, sterile cefuroxime sodium, per 750 mg
HCPC	J0698	Injection, cefotaxime sodium, per gm
HCPC	J2510	Injection, penicillin g procaine, aqueous, up to 600,000 units
HCPC	J2540	Injection, penicillin g potassium, up to 600,000 units

Appendix Table 3. ICD-10-CM codes used to identify disseminated LD manifestations

Type	Category	Code	Description
Musculoskeletal	Pain in joint	M25.561	Pain in right knee
Musculoskeletal	Pain in joint	M25.562	Pain in left knee
Musculoskeletal	Arthritis and joint effusion	M00.80	Arthritis due to other bacteria, unspecified joint
Musculoskeletal	Arthritis and joint effusion	M00.869	Arthritis due to other bacteria, unspecified knee
Musculoskeletal	Arthritis and joint effusion	M00.88	Arthritis due to other bacteria, vertebrae
Musculoskeletal	Arthritis and joint effusion	M00.89	Polyarthritis due to other bacteria
Musculoskeletal	Arthritis and joint effusion	M13.10	Monoarthritis, not elsewhere classified, unspecified site
Musculoskeletal	Arthritis and joint effusion	M13.161	Monoarthritis, not elsewhere classified, right knee
Musculoskeletal	Arthritis and joint effusion	M13.162	Monoarthritis, not elsewhere classified, left knee
Musculoskeletal	Arthritis and joint effusion	M13.169	Monoarthritis, not elsewhere classified, unspecified knee
Musculoskeletal	Arthritis and joint effusion	M25.40	Effusion, unspecified joint
Musculoskeletal	Arthritis and joint effusion	M25.461	Effusion, right knee
Musculoskeletal	Arthritis and joint effusion	M25.462	Effusion, left knee
Musculoskeletal	Arthritis and joint effusion	M25.469	Effusion, unspecified knee
Musculoskeletal	Arthritis and joint effusion	M25.48	Effusion, other site
Musculoskeletal	Arthropathy	M01.X0	Direct infection of unspecified joint in infectious and parasitic diseases classified elsewhere
Musculoskeletal	Arthropathy	M01.X6	Direct infection of knee in infectious and parasitic diseases classified elsewhere
Musculoskeletal	Arthropathy	M01.X9	Direct infection of multiple joints in infectious and parasitic diseases classified elsewhere
Musculoskeletal	Arthropathy	M01.X61	Direct infection of right knee in infectious and parasitic diseases classified elsewhere
Musculoskeletal	Arthropathy	M01.X62	Direct infection of left knee in infectious and parasitic diseases classified elsewhere
Musculoskeletal	Arthropathy	M01.X69	Direct infection of unspecified knee in infectious and parasitic diseases classified elsewhere
Musculoskeletal	Arthropathy	M01.X79	Direct infection of unspecified ankle and foot in infectious and parasitic diseases classified elsewhere
Musculoskeletal	Arthropathy	M01.X8	Direct infection of vertebrae in infectious and parasitic diseases classified elsewhere
Musculoskeletal	Arthropathy	M02.80	Other reactive arthropathies, unspecified site
Musculoskeletal	Arthropathy	M02.861	Other reactive arthropathies, right knee
Musculoskeletal	Arthropathy	M02.862	Other reactive arthropathies, left knee
Musculoskeletal	Arthropathy	M02.869	Other reactive arthropathies, unspecified knee
Musculoskeletal	Arthropathy	M02.88	Other reactive arthropathies, vertebrae
Musculoskeletal	Arthropathy	M02.89	Other reactive arthropathies, multiple sites
Neurologic	Meningitis	G00.8	Other bacterial meningitis
Neurologic	Meningitis	G00.9	Bacterial meningitis, unspecified
Neurologic	Meningitis	G01	Meningitis in bacterial diseases classified elsewhere
Neurologic	Meningitis	G03.0	Nonpyogenic meningitis
Neurologic	Meningitis	G03.9	Meningitis, unspecified
Neurologic	Meningitis	G04.2	Bacterial meningoencephalitis and meningomyelitis, not elsewhere classified
Neurologic	Bell's/Facial Palsy	G51.0	Bell's palsy
Neurologic	Bell's/Facial Palsy	G51.8	Other disorders of facial nerve
Neurologic	Bell's/Facial Palsy	G51.9	Disorder of facial nerve, unspecified
Neurologic	Bell's/Facial Palsy	G52.7	Disorders of multiple cranial nerves
Neurologic	Bell's/Facial Palsy	G52.8	Disorders of other specified cranial nerves
Neurologic	Bell's/Facial Palsy	G52.9	Cranial nerve disorder, unspecified
Neurologic	Bell's/Facial Palsy	G53	Cranial nerve disorders in diseases classified elsewhere
Neurologic	Bell's/Facial Palsy	G56.00	Carpal tunnel syndrome, unspecified upper limb
Neurologic	Bell's/Facial Palsy	G56.01	Carpal tunnel syndrome, right upper limb
Neurologic	Bell's/Facial Palsy	G56.02	Carpal tunnel syndrome, left upper limb
Neurologic	Bell's/Facial Palsy	G56.03	Carpal tunnel syndrome, bilateral upper limbs
Neurologic	Bell's/Facial Palsy	G57.81	Other specified mononeuropathies of right lower limb
Neurologic	Bell's/Facial Palsy	G57.82	Other specified mononeuropathies of left lower limb
Neurologic	Bell's/Facial Palsy	G57.83	Other specified mononeuropathies of bilateral lower limbs
Neurologic	Bell's/Facial Palsy	G57.90	Unspecified mononeuropathy of unspecified lower limb
Neurologic	Bell's/Facial Palsy	G57.91	Unspecified mononeuropathy of right lower limb
Neurologic	Bell's/Facial Palsy	G57.92	Unspecified mononeuropathy of left lower limb
Neurologic	Bell's/Facial Palsy	G57.93	Unspecified mononeuropathy of bilateral lower limbs
Neurologic	Bell's/Facial Palsy	G58.0	Intercostal neuropathy
Neurologic	Bell's/Facial Palsy	G58.7	Mononeuritis multiplex
Neurologic	Bell's/Facial Palsy	G58.8	Other specified mononeuropathies
Neurologic	Bell's/Facial Palsy	G58.9	Mononeuropathy, unspecified
Neurologic	Bell's/Facial Palsy	G59	Mononeuropathy in diseases classified elsewhere

Type	Category	Code	Description
Neurologic	Bell's/Facial Palsy	R29.810	Facial weakness
Neurologic	Bell's/Facial Palsy	S04.50XA	Injury of facial nerve, unspecified side, initial encounter
Neurologic	Bell's/Facial Palsy	S04.51XA	Injury of facial nerve, right side, initial encounter
Neurologic	Bell's/Facial Palsy	S04.52XA	Injury of facial nerve, left side, initial encounter
Neurologic	Bell's/Facial Palsy	S04.891A	Injury of other cranial nerves, right side, initial encounter
Neurologic	Bell's/Facial Palsy	S04.892A	Injury of other cranial nerves, left side, initial encounter
Neurologic	Bell's/Facial Palsy	S04.899A	Injury of other cranial nerves, unspecified side, initial encounter
Neurologic	Radiculopathy	M54.1	Radiculopathy
Neurologic	Radiculopathy	M54.10	Radiculopathy, site unspecified
Neurologic	Radiculopathy	M54.11	Radiculopathy, occipito-atlanto-axial region
Neurologic	Radiculopathy	M54.12	Radiculopathy, cervical region
Neurologic	Radiculopathy	M54.13	Radiculopathy, cervicothoracic region
Neurologic	Radiculopathy	M54.14	Radiculopathy, thoracic region
Neurologic	Radiculopathy	M54.15	Radiculopathy, thoracolumbar region
Neurologic	Radiculopathy	M54.16	Radiculopathy, lumbar region
Neurologic	Radiculopathy	M54.17	Radiculopathy, lumbosacral region
Neurologic	Radiculopathy	M54.18	Radiculopathy, sacral and sacrococcygeal region
Neurologic	Encephalitis	A84.8	Other tick-borne viral encephalitis
Neurologic	Encephalitis	A84.89	Other tick-borne viral encephalitis
Neurologic	Encephalitis	A84.9	Tick-borne viral encephalitis, unspecified
Neurologic	Encephalitis	G04.90	Encephalitis and encephalomyelitis, unspecified
Neurologic	Polyneuropathy	G61.9	Inflammatory polyneuropathy, unspecified
Neurologic	Polyneuropathy	G62.9	Polyneuropathy, unspecified
Neurologic	Polyneuropathy	G63	Polyneuropathy in diseases classified elsewhere
Neurologic	Nerve root and plexus disorders	G54.2	Cervical root disorders, not elsewhere classified
Neurologic	Nerve root and plexus disorders	G54.3	Thoracic root disorders, not elsewhere classified
Neurologic	Nerve root and plexus disorders	G54.4	Lumbosacral root disorders, not elsewhere classified
Neurologic	Mononeuropathies of lower limb	G57.0	Lesion of sciatic nerve
Neurologic	Mononeuropathies of lower limb	G57.00	Lesion of sciatic nerve, unspecified lower limb
Neurologic	Mononeuropathies of lower limb	G57.01	Lesion of sciatic nerve, right lower limb
Neurologic	Mononeuropathies of lower limb	G57.02	Lesion of sciatic nerve, left lower limb
Neurologic	Mononeuropathies of lower limb	G57.03	Lesion of sciatic nerve, bilateral lower limbs
Neurologic	Mononeuropathies of lower limb	G57.8	Other specified mononeuropathies of lower limb
Cardiac	Myocarditis and pericarditis	I30.0	Acute nonspecific idiopathic pericarditis
Cardiac	Myocarditis and pericarditis	I30.8	Other forms of acute pericarditis
Cardiac	Myocarditis and pericarditis	I30.9	Acute pericarditis, unspecified
Cardiac	Myocarditis and pericarditis	I32	Pericarditis in diseases classified elsewhere
Cardiac	Myocarditis and pericarditis	I40.0	Infective myocarditis
Cardiac	Myocarditis and pericarditis	I40.8	Other acute myocarditis
Cardiac	Myocarditis and pericarditis	I40.9	Acute myocarditis, unspecified
Cardiac	Myocarditis and pericarditis	I41	Myocarditis in diseases classified elsewhere
Cardiac	Myocarditis and pericarditis	I51.4	Myocarditis, unspecified
Cardiac	Myocarditis and pericarditis	I45.4	Nonspecific intraventricular block
Cardiac	Myocarditis and pericarditis	I45.5	Other specified heart block
Cardiac	AV Block	I44.0	Atrioventricular block, first degree
Cardiac	AV Block	I44.1	Atrioventricular block, second degree
Cardiac	AV Block	I44.2	Atrioventricular block, complete
Cardiac	AV Block	144.3	Other and unspecified atrioventricular block
Cardiac	AV Block	I44.30	Unspecified atrioventricular block
Cardiac	AV Block	I44.39	Other atrioventricular block
Cardiac	AV Block	I45.89	Other specified conduction disorders
Cardiac	AV Block	I45.9	Conduction disorder, unspecified

Appendix Table 4. Percent clinical characteristics of LD cases by age group and race ethnicity group, US Medicaid ≤18 y old, 2016–2021*

Characteristic	White, n = 29,405	Black, n = 1,244	Asian, Pacific Islander; n = 741	Hispanic, n = 2,063	Native American, n = 208
Disease stage and manifestations					
Localized	84.1	60.9	75.6	75.7	78.8
Disseminated disease	15.9	39.1	24.4	24.3	21.2
Neurologic	26.6	17.9	–	–	–
Musculoskeletal (arthritis)	71.6	79.3	84	72.9	63.6
Cardiac	1.8	2.9	–	–	–
Hospitalization at diagnosis	2.2	6.1	3.4	4	–
Diagnosis in primary care	66.1	62.7	52.1	66.9	41.3
Diagnosis during October–March	41.4	54.9	48.4	49.8	43.8
Sex					
M	54.2	54.7	54.1	49.7	57.7
F	44.8	45.3	45.9	50.3	42.3
Mean CCI (SD)	1.06 (0.34)	1.04 (0.24)	1.04 (0.21)	1.04 (0.23)	1.13 (0.52)
Mean visits before LD diagnosis (SD)					
30 d before	1.8 (2.49)	2.05 (2.58)	1.86 (2.5)	1.98 (2.19)	2.04 (3.1)
60 d before	3.16 (4.54)	3.43 (4.64)	3.06 (4.38)	3.41 (3.82)	3.42 (5.08)
183 d before	8.75 (12.78)	8.82 (13.05)	7.80 (12.47)	8.99 (10.57)	9.12 (13.74)
Antibiotics prescribed					
Amoxicillin	57.8	53.8	63.0	61.7	48.1
Doxycycline	43.2	48.2	39.1	40.8	52.9
Laboratory testing CPT 86618: *Borrelia burgdorferi*	14.1	19.7	20.1	21.8	28.4

*Values are %, except where otherwise indicated. CCI, Charlson Comorbidity index; –, sample size <11, thus counts suppressed from calculations.

Appendix Table 5. Percent clinical characteristics of LD cases by age group and race ethnicity group, US Medicaid ≥19 y old, 2016–2021*

Characteristic	White, n = 26,298	Black, n = 1,461	Asian, Pacific Islander; n = 1,082	Hispanic, n = 1,867	Native American, n = 191
Disease stage and manifestations					
Localized	82.7	70.4	78.7	70.6	77.5
Disseminated disease	17.3	29.6	21.3	29.4	22.5
Neurologic	45.2	47	–	–	–
Musculoskeletal (arthritis)	50.6	48.8	59.6	59.9	51.2
Cardiac	4.2	4.2	–	–	–
Hospitalization at diagnosis	4.3	7.4	3.0	4.4	–
Diagnosis in primary care	61.6	57.1	51.1	65.6	57.1
Diagnosis during October–March	49.2	62.1	52.7	60.4	45.6
Sex					
M	45.9	37	37.9	30.8	47.1
F	54.1	63	62.1	69.2	52.9
Mean CCI (SD)	1.66 (1.31)	1.93 (1.61)	1.70 (1.37)	1.72 (1.47)	2.04 (1.7)
Mean visits before LD diagnosis (SD)					
30 d before	2.73 (3.42)	3.45 (3.75)	2.75 (3.05)	3.16 (3.75)	3.2 (4.42)
60 d before	4.94 (6.33)	6.13 (6.72)	4.77 (5.58)	5.59 (6.79)	5.87 (8.47)
183 d before	13.62 (17.63)	15.43 (17.28)	12.51 (15.01)	14.44 (17.67)	15.51 (22.42)
Antibiotics prescribed					
Amoxicillin	15.7	20.6	16.6	20.4	15.2
Doxycycline	85.6	81.2	86.8	81.0	89.0
Laboratory testing CPT 86618: *Borrelia burgdorferi*	15.3	19.4	18.9	25.2	28.8

*Values are %, except where otherwise indicated. CCI, Charlson Comorbidity index; –, sample size <11, thus counts suppressed from calculations.

Appendix Table 6. Percent clinical characteristics of LD cases by age group and race ethnicity group, US Medicare <65 y old, 2016–2021*

Characteristic	White, n = 11,845	Black, n = 513	Asian, Pacific Islander; n = 72	Hispanic, n = 293	Native American, n = 53
Disease stage and manifestations					
Localized	71.8	57.3	76.4	67.2	69.8
Disseminated disease	28.2	42.7	23.6	32.8	30.2
Neurologic	64.4	62.1	–	58.3	75
Musculoskeletal (arthritis)	32.9	35.2	–	39.6	–
Cardiac	2.7	–	–	–	–
Hospitalization at diagnosis	8.2	12.5	–	7.2	–
Diagnosis in primary care	60.7	48.5	58.3	54.6	66
Diagnosis during October–March	55.8	63.0	52.8	59.7	60.4
Sex					
M	43.9	31.8	34.7	37.9	43.4
F	56.1	68.2	65.3	62.1	56.6
Mean CCI (SD)	2.44 (1.97)	3.28 (2.5)	2.79 (2.36)	2.57 (1.81)	2.31 (1.97)
Mean visits before LD diagnosis (SD)					
30 d before	0.99 (1.52)	1.17 (1.67)	0.93 (1.4)	0.91 (1.42)	1.38 (1.55)
60 d before	1.75 (2.5)	2.04 (2.72)	1.60 (2.51)	1.71 (2.41)	2.28 (2.35)
183 d before	4.76 (6.19)	5.44 (6.43)	4.38 (6.39)	4.75 (6.02)	6.28 (6.20)
Antibiotics prescribed					
Amoxicillin	19.4	27.5	22.2	29.7	–
Doxycycline	77.7	69.6	69.4	73.7	90.6
Laboratory testing CPT 86618: *Borrelia burgdorferi*	47.4	48.7	41.7	50.9	50.9

*Values are %, except where otherwise indicated. CCI, Charlson Comorbidity index; –, sample size <11, thus counts suppressed from calculations.

Appendix Table 7. Percent clinical characteristics of LD cases by age group and race ethnicity group, US Medicare ≥65 y old, 2016–2021*

Characteristic	White, n = 87,831	Black, n = 1,172	Asian, Pacific Islander; n = 591	Hispanic, n = 293	Native American, n = 64
Disease stage and manifestations					
Localized	79.2	69.4	74.3	69.6	76.6
Disseminated disease	20.8	30.6	25.7	30.4	23.4
Neurologic	53.7	54.3	–	52.3	–
Musculoskeletal (arthritis)	39	42.1	48	–	–
Cardiac	7.3	3.6	–	–	–
Hospitalization at diagnosis	7.2	7.7	8.1	8.5	–
Diagnosis in primary care	65.2	52.3	52.6	50.9	78.1
Diagnosis during October–March	48.7	59.1	50.6	62.5	56.3
Sex					
M	47.2	40.6	41.8	33.9	54.7
F	52.8	59.4	58.2	66.1	45.3
Mean CCI (SD)	2.58 (1.99)	3.09 (2.27)	2.38 (1.86)	3.09 (2.41)	3.2 (2.14)
Mean visits before LD diagnosis (SD)					
30 d before	0.71 (1.21)	0.62 (1.04)	0.42 (0.88)	0.62 (1.12)	1.2 (1.51)
60 d before	1.21 (1.88)	1.05 (1.69)	0.66 (1.28)	1.03 (1.74)	2.05 (2.49)
183 d before	3.13 (4.41)	2.81 (4.11)	1.54 (2.38)	2.89 (4.34)	5.69 (6.89)
Antibiotics prescribed					
Amoxicillin	15.5	21.5	22.2	24	–
Doxycycline	83.7	77.2	78.7	68.6	89.1
Laboratory testing CPT 86618: *Borrelia burgdorferi*	48.1	53.6	56.0	50.2	48.4

*Values are %, except where otherwise indicated. CCI, Charlson Comorbidity index; –, sample size <11, thus counts suppressed from calculations.

	N	Jan	Feb	March	April	May	June	July	Aug	Sept	Oct	Nov	Dec
**Medicaid ≤18**													
White	29,405	2.0%	1.8%	2.4%	3.7%	8.4%	20.5%	24.7%	13.4%	8.1%	7.1%	5.1%	2.9%
Black	1244	4.7%	3.2%	4.0%	5.4%	10.3%	15.2%	16.6%	13.3%	9.4%	7.9%	5.8%	4.3%
Asian/Pacific Islander	741	3.2%	2.4%	4.3%	4.6%	8.9%	16.5%	22.3%	12.8%	9.6%	7.3%	5.4%	2.7%
Hispanic	208	3.3%	3.2%	4.0%	4.5%	9.2%	15.8%	21.0%	13.4%	8.0%	7.2%	6.1%	4.3%
**Medicaid ≥19**													
White	26,298	3.0%	2.7%	3.4%	4.9%	8.3%	15.7%	21.5%	13.6%	8.0%	8.1%	6.8%	4.0%
Black	1461	5.4%	4.7%	5.5%	6.6%	9.8%	11.1%	14.5%	12.3%	8.4%	9.7%	6.9%	5.1%
Asian/Pacific Islander	1082	3.7%	2.6%	4.1%	5.0%	9.0%	14.1%	19.0%	14.1%	9.1%	8.3%	5.7%	5.3%
Hispanic	1867	4.8%	4.6%	5.4%	7.1%	8.1%	11.8%	15.6%	12.2%	9.1%	8.4%	7.4%	5.6%
**Medicare <65**													
White	11,845	4.4%	4.0%	5.1%	6.3%	10.2%	15.5%	17.1%	11.6%	7.3%	7.8%	6.7%	4.0%
Black	513	5.3%	5.8%	6.8%	8.6%	9.2%	14.0%	13.5%	9.6%	8.6%	6.8%	8.4%	3.5%
Hispanic	293	4.8%	3.8%	5.8%	7.5%	9.2%	15.7%	13.3%	11.3%	10.6%	7.9%	6.1%	4.1%
**Medicare ≥65**													
White	87,831	2.8%	2.3%	3.1%	5.6%	10.5%	18.6%	20.3%	12.4%	7.2%	7.2%	6.3%	3.7%
Black	1172	5.1%	3.2%	5.5%	7.4%	9.1%	14.7%	15.8%	10.4%	9.4%	8.8%	6.1%	4.4%
Asian/Pacific Islander	591	3.9%	1.9%	4.4%	5.2%	10.7%	17.1%	16.6%	15.7%	9.3%	6.3%	5.6%	3.4%
Hispanic	283	7.1%	0.0%	5.3%	7.1%	10.6%	12.7%	17.0%	7.8%	7.8%	7.4%	8.8%	0.0%

Appendix Figure. Percentage of LD cases by race/ethnicity group and month for beneficiaries of U.S. Medicaid and Medicare identifying as White, Black, Asian/Pacific Islander, or Hispanic 2016–2021. Data not displayed for beneficiaries of Medicare aged <65 who identified as Asian/Pacific Islander because of small sample size.
